# Novel Precursor for h‑BN Synthesis on Ni(111) Substrates

**DOI:** 10.1021/acs.jpcc.5c03822

**Published:** 2025-08-21

**Authors:** Sergi Campos-Jara, Tycho Roorda, Laurens P. M. de Jong, Vladyslav Virchenko, Andy Jiao, Mauricio J. Prieto, Liviu C. Tanase, Mohamad A. Mawass, Jing-Wen Hsueh, Vladimir Calvi, Jetse van Os, Núria Félez-Guerrero, Rick Monsma, Richard van Rijn, Thomas Schmidt, Grégory Schneider, Irene M. N. Groot

**Affiliations:** † Leiden Insitute of Chemistry, 4496 Leiden University, Einsteinweg 55, Leiden 2333 CC, Netherlands; ‡ Department of Interface Science, 28259 Fritz-Haber-Institut der Max-Planck-Gesellschaft, Faradayweg 4-6, Berlin 14195, Germany; § Applied Nanolayers, Delft University of Technology, Feldmanweg 17, Delft 2638 CT, Netherlands

## Abstract

In this study, we report the synthesis of single-crystalline h-BN on Ni(111) under ultrahigh vacuum (UHV) conditions using hexamethylborazine (HMB) as a nonclassical precursor. The novel use of HMB facilitates the diffusion of methyl groups into the bulk of Ni(111), playing a critical role in the achievement of high-quality crystalline h-BN layers. The synthesis is performed on a 2 mm-thick Ni(111) single crystal and on a 2-μm-thick Ni(111) thin film on sapphire to evaluate the feasibility of synthesizing h-BN on industrially relevant substrates. Advanced microscopic and spectroscopic techniques confirm the successful synthesis of h-BN. The growth of h-BN was investigated by scanning tunneling microscopy and low-energy electron microscopy. Low-energy electron diffraction confirms the single crystallinity of the grown 2-dimensional layer. X-ray photoelectron spectroscopy confirms the presence of boron and nitrogen bonds at the same binding energies reported in the literature for h-BN. In contrast, photoemission electron microscopy allows identification of the presence of h-BN throughout the Ni(111) surface. This work advances the understanding of h-BN growth mechanisms on metal substrates and provides a foundation for improving synthesis methods to meet the demands of next-generation materials and devices.

## Graphical abstract

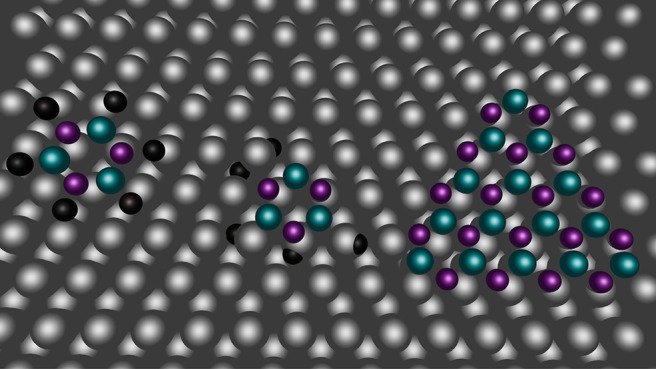

## Introduction

Over the past 20 years, since the experimental discovery of graphene in 2004,^1^ scientific interest in two-dimensional (2D) materials has grown significantly. This led to the discovery of different kinds of 2D materials: inorganic (graphene, hexagonal boron nitride (h-BN), borophene etc.), transition metal dichalcogenides (e.g., MoS_2_, WS_2_), or non-noble metals (2D Ga, 2D In, etc.).^2^ 2D materials have gained substantial interest due to their tunable bandgap, surface and edge reactivity, and unique electronic and optoelectronic properties, among others.^3–6^ Their unique 2D structure opens a wide range of opportunities for customizing these layers through various techniques such as exploring the number of layers, novel synthesis routes, engineering defects, morphology control, or moiré engineering.^7,8^

Focusing on the inorganic 2D materials, h-BN is isostructural to graphene with an sp^2^ hybridization of alternating boron (B) and nitrogen (N) atoms organized in a honeycomb structure of a 2.46 Å lattice parameter.^9^ Despite its structural similarities with graphene, h-BN has antagonistic electronic properties with a large bandgap of 5–6 eV making h-BN an insulator.^10^ h-BN’s large bandgap induces a lack of electrical conductivity; however, its high thermal conductivity makes h-BN a useful material for electronic devices.^11,12^ h-BN has also proven to work as an ultraviolet light emitter in optoelectronic devices and as a nanofiller in high-strength and thermally conductive nanocomposites.^13–16^ Furthermore, h-BN is chemically inert and has proven to be resistant to oxidation under diverse conditions becoming a compelling material for various types of coatings.^17^

As a result of the growing interest, many research efforts have been devoted toward growing large-area, high-quality h-BN.^18^ A wide range of methods have been employed to grow h-BN, from powder production, as bulk crystals, and as thin layers on nonmetallic substrates (e.g., Al_2_O_3_) (via molecular beam epitaxy (MBE)), to a wide range of metallic substrates such as Cu, Ni, Co, and Rh (via MBE, plasma-assisted growth, or chemical vapor deposition (CVD)).^19–21^ So far, CVD has proven to be one of the best methods for h-BN growth, employing borazine and ammonia borane as the most common precursors.^22–25^ Typically, the substrate is annealed at high temperatures (1200 K) to induce precursor decomposition on the surface upon its dosing on the substrate, thereby growing the h-BN on the (metallic) surface.^19–25^ Growing h-BN via CVD on metal surfaces (compared to top-down approaches) has shown significant advantages, especially in terms of the thickness and quality of the h-BN film.^18^ Furthermore, the size of the grown film in the 2D plane, in principle, depends only on the size of the substrate. However, despite all the improvements in h-BN growth, synthesis using most of these methods still has some major limitations. The high temperatures required for h-BN growth and the difficulties in handling the precursors are two of the major challenges.^26–28^

Ammonia borane and borazine tend to polymerize at high temperatures when used in large quantities making their removal from the CVD reactor equipment (i.e., crucibles, pumps, reactor walls) more demanding. Additionally, borazine must be stored at low temperatures (<−20 °C) complicating the synthesis procedure and the equipment used for evaporation. Furthermore, the main side product of h-BN growth with these precursors is hydrogen (H_2_), which in large quantities requires safety equipment like gas detectors, water sprinklers, or gas neutralizers.^29^ These constraints make scaling up the growth of h-BN expensive; thus, it is hard to compete with cheaper materials, despite its outstanding properties.

Aiming to mitigate these challenges and enable a scalable production method for 2D materials, we report the synthesis of single-crystalline h-BN on Ni(111) using an alternative precursor. The synthesis is performed on a Ni(111) 2 mm-thick single crystal (Ni(111) SC) and a 2 μm-thick Ni(111) thin film on a sapphire substrate (Ni(111) TF), allowing us to establish a direct correlation between a susbtrate used for fundamental research (Ni(111) SC) and a typical industrial susbtrate for the growth of h-BN (Ni(111) TF). The synthesis of h-BN is performed using a nonconventional precursor, namely hexamethylborazine (HMB, C_6_H_18_N_3_B_3_). The steps for the synthesis of HMB are reported in Figures S1–S4 for nuclear magnetic resonance characterization of the different steps. HMB is deposited onto Ni(111) via CVD at relatively low temperatures (823 K, 550 °C) in ultrahigh vacuum (UHV) to grow h-BN. Due to the high Ni(111) catalytic activity,^30^ it allows for a more efficient demethylation and, therefore, the formation of h-BN at lower synthesis temperature. The use of HMB as a BN precursor also reduces the formation of H_2_ and the polymerization of the molecule, which occur at high temperatures.

## Experimental Methods

The characterization of the Ni(111) TF was performed at our laboratory in Leiden University, while the characterization of the Ni(111) SC was performed at the SMART beamline at BESSY II, Germany.

### Ni­(111) Cleaning Procedure

Both Ni(111) substrates were first annealed at 823 K for 5 min under 10^–6^ mbar of O_2_ atmosphere. Subsequently, the substrates were cleaned by performing multiple Ar^+^ sputtering cycles at 10^–6^ mbar for 15 min at room temperature followed by annealing at 823 K for 10 min.

### Hexamethylborazine Synthesis H-BN Growth

The same synthesis procedure was performed on both the Ni(111) SC and the Ni(111) TF. The substrates were annealed at 823 K, and once this temperature was reached, the substrates were exposed for 15 min to 10^–6^ mbar of HMB. The deposition was followed using a mass spectrometer.

### Scanning Tunneling Microscopy (STM)

STM was performed at room temperature using the UHV mode of the ReactorSTM.^31^ Tips were prepared by cutting a polycrystalline Pt–Ir 90–10 wire purchased from Goodfellow without further processing. Constant current scans were performed using video-rate scanning electronics described in detail elsewhere.^32,33^ Image processing was performed using WSxM software.^34^ The most common filtering was used to obtain a correctly treated surface, including flattening, Gaussian smoothing, and local plane. For the atomic resolution image (Figure 1b), a 2DFFT (2-dimensional fast Fourier transform) was used to select the desired information and remove the noise. The original image can be seen in Figure S5. No further processing was performed on the images reported in this work.

**Figure 1:**
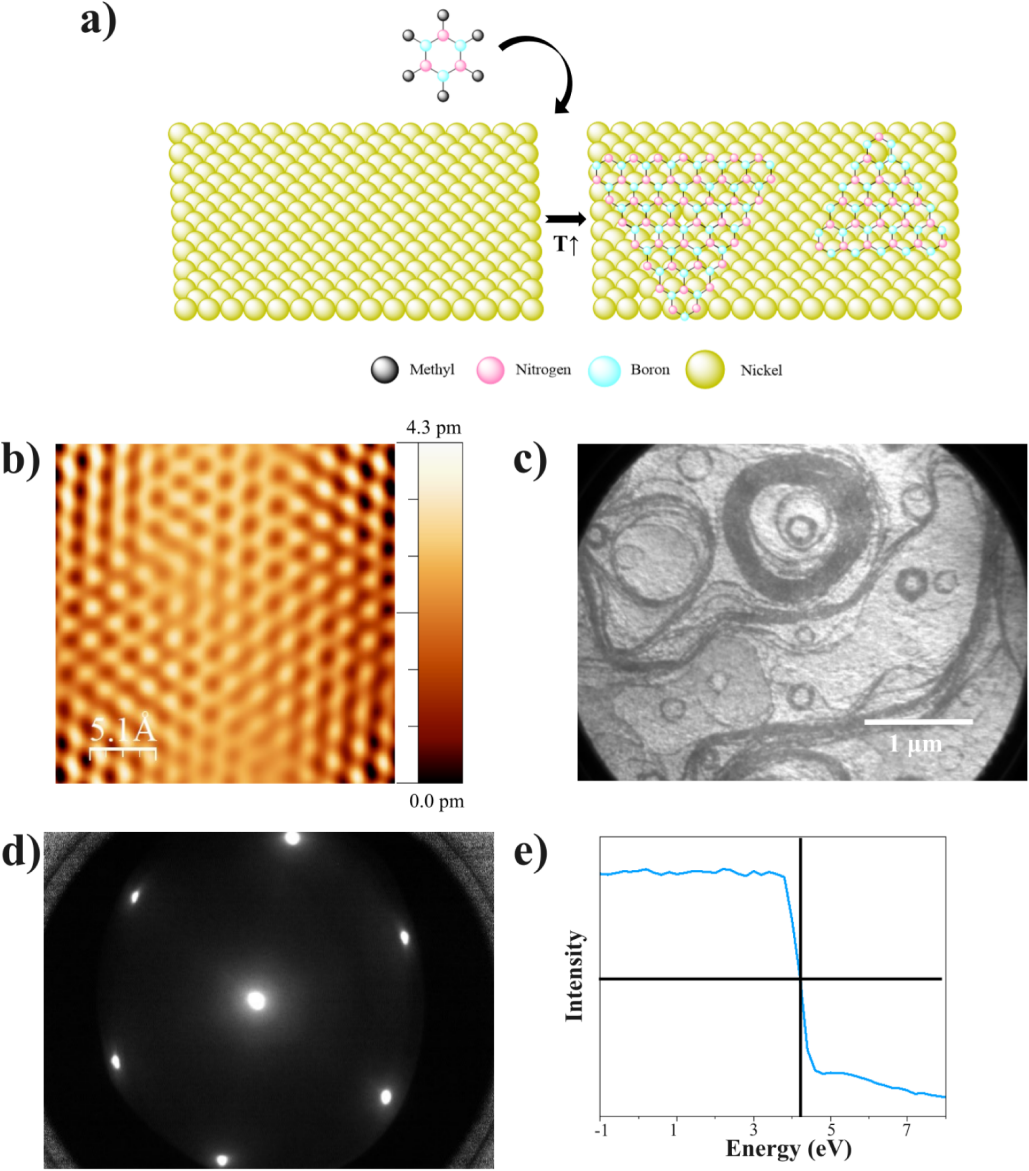
h-BN on Ni(111) microscopy and diffraction characterization. (a) Schematics of the reaction procedure. (b) STM image. 2.5 × 2.5 nm^2^, *I*_t_ = 65 pA and *V*_Bias_ = −4.7 V. (c) 4.27 μm of the lens aperture LEEM image of the h-BN film. Energy = 5 eV, scale bar = 1 μm. (d) LEED pattern at 42 eV. (e) Work function calculation extracted from the LEEM-IV characterization (−4 to 5 eV).

### X-ray Photoelectron Spectroscopy (XPS)

The XPS measurements were performed in a SPECS Phoibos system equipped with an XRM50 X-ray source set to the Al K-alpha line, used along with a monochromator to excite the sample with a beam spot of 0.4 mm diameter at 55° incidence. The acceleration voltage was set to 12 kV and a power of 400 W was used for all of the measurements. The HSA3500 hemispherical analyzer with a pass energy of 20 eV was employed to analyze the photoemission.

All the XPS data were analyzed using CASA XPS. Shirley and Tougaard’s backgrounds were subtracted from the different binding energy regions of the elements. All XPS spectra were calibrated relative to the Ni 2p peak. The peak areas were corrected for their relative sensitivity factors for atomic concentrations’ calculations.^35^

### Mass Spectrometry

A PrismaPlus mass spectrometer from Pfeiffer Vacuum has been used to confirm the presence of hexamethylborazine in the UHV chamber during the deposition. During evaporation, the mass spectrometer was used to confirm the presence of HMB by comparing it with the reference data from the NIST database. Before the deposition of hexamethylborazine, mass spectrometry was employed to confirm the cleanliness of the ultrahigh vacuum (UHV) environment. This step was essential to ensure that the mass spectrometric readings corresponded to those expected in a standard UHV setup, free of any extraneous gases or contaminants.

### Low-Energy Electron Diffraction (LEED)

A SPECS ErLEED 100/150 instrument with ErLEED 3000D electronics was employed for the LEED diffraction pattern of the Ni(111) thin film. The diffraction patterns were taken at 80, 90, and 100 eV. The LEED pictures were taken with an EOS 4000D camera, using a low-light exposure. They were enhanced by increasing the contrast and the saturation.

### Measurements Performed at BESSY II: Low-Energy Electron Microscopy (LEEM), Low-Energy Electron Diffraction (LEED), X-ray Photoelectron Spectroscopy (XPS), Photoemission Electron Microscopy (PEEM), and Angle-Resolved Photoelectron Spectroscopy (ARPES)

The combined LEEM/XPEEM, LEED/ARPES, and XPS experiments were carried out using the SMART spectromicroscope operating at the UE49-PGM beamline of the synchrotron light source BESSY-II of the Helmholtz Centre Berlin for Materials and Energy (HZB). The aberration-corrected and energy-filtered instrument, which has been described in detail elsewhere^36,37^ combines microscopy (LEEM/XPEEM), low-energy electron diffraction (μ-LEED), and laterally resolved X-ray spectroscopy (μ-XPS). The SMART microscope achieves a maximal lateral resolution of 2.6 and 18 nm in LEEM and XPEEM modes, respectively.^38,39^ The system is equipped with gas dosing (Ar, H_2_, and O_2_; purity 99.999%), an Ar sputter gun for sample cleaning, and evaporators for thin-film deposition.

In LEEM, the low-energy electrons elastically backscattered from the sample surface are exploited for imaging, whereby the bright-field imaging mode with a contrast aperture selecting the specularly reflected electron beam was used. In XPEEM, the sample surface is illuminated with X-rays and photoemitted electrons are used for imaging. The imaging energy analyzer allows the selection of electrons having a binding energy within a window and their use to form the XPEEM image. The choice of energy windows corresponding to the XPS peaks specific for particular elements and their chemical states provides chemical contrast. In LEED the sample surface is illuminated like in LEEM and the elastically backscattered electrons are detected. However, whereas in LEEM the intermediate image plane is imaged onto the two-dimensional detector, in LEED mode, the intermediate back focal plane is imaged onto the detector showing the diffraction pattern of the electrons. By inserting small apertures into the intermediate image plane, one can select the surface area from which the LEED pattern is obtained (μ-LEED). Because this imaging is done through the imaging energy analyzer, the inelastically backscattered electrons are cut away, and only the elastically reflected electrons are detected. For ARPES, the same setup is used; however, this time the sample surface is not illuminated with an electron beam but with an X-ray beam, and the photoemitted electrons are detected. Because of the identical electron optical setup, the k-space calibration from LEED can be transferred to the ARPES.

## Results and Discussion

The clean Ni(111) surface was characterized using low-energy electron microscopy (LEEM) and low-energy electron diffraction (LEED) (see Figure S6a,b). Both Ni(111) substrates were cleaned by performing cycles of sputtering of Ar^+^ (1 keV, 1 × 10^–6^ mbar) for 15 min with subsequent annealing ≥823 K for 10 min.

The same growth procedure was employed for both Ni(111) and Ni(111) substrates. Figure 1a presents a mechanistic representation of the growth. The clean Ni(111) substrates were annealed at 823 K. Subsequently, the substrates were exposed to 2 × 10^–6^ mbar of HMB for 15 min. The atomic-resolution scanning tunneling microscopy (STM) image shown in Figure 1b shows a honeycomb structure typical of the atomic arrangement of h-BN grown on the Ni(111) TF. The interatomic distance is calculated to be 1.45 ± 0.17 Å, which is in good agreement with the values reported for h-BN grown on Ni(111) using ammonia borane and borazine, respectively.^40,41^ The morphology of the surface was characterized using LEEM. The contrast in LEEM is principally produced by the interaction of low-energy electrons with the topmost atomic layers on the surface. Such an interaction is translated in a change in the electron reflectivity (*R*(*E*)). As observed in the LEEM image (see Figure 1c), the surface after HMB deposition appears rougher than the clean surface (see Figure S6) indicating the deposition of the molecule or the growth of h-BN on the surface of the Ni(111) SC. The LEEM images in Figure 1c and the ones taken with larger fields of view (Figure S6a) are in good agreement with the LEEM characterization performed by Basu et al. for h-BN synthesis on Cu(111) and Lu et al. for h-BN synthesis on a Cu–Ni alloy.^42,43^ The LEED pattern reported in Figure 1d (see Figure S7b for LEED patterns at higher energies) shows the diffraction pattern for the h-BN grown on Ni(111) SC, where only the (111) spots are visible, as it was for the clean Ni(111) LEED patterns (Figure S6b). The same result is obtained when LEED characterization is performed on the h-BN grown on the Ni(111) TF (see Figure S8). This could be explained by two factors: h-BN on Ni(111) mainly grows in single-crystalline domains, as reported by Ma et al.^20^ Additionally, Ni(111) and h-BN have a small lattice mismatch of less than 0.4% as reported by Islam et al.^21^ The growth of h-BN on substrates with similar lattice constants tends to be commensurate and the h-BN lattice will be extended to match the lattices of Ni(111).^44,45^ Therefore, the (111) LEED structure shows overlapping spots of both the lattice of Ni and h-BN.^21,46^ Additionally, LEEM-IV (intensity vs voltage) was performed to determine the work function (Φ) of the h-BN grown on the Ni(111) SC as shown in Figure 1e.^47^ The measured Φ through LEEM-IV is Φ = 4.1 eV, a significant reduction of the work function compared to clean Ni(111) (Φ = 5.3–5.6 eV).^48^ These measurements are in good agreement with the experimental results reported in the literature, where the work function for h-BN on top of metal substrates is around 4.2–4.4 eV.^49^

The synthesis of h-BN requires the formation of B–N bonds to interconnect the HMB molecules, forming a continuous film. This was investigated on both substrates via X-ray photoelectron spectroscopy (XPS). Figure 2a,b show the N 1s and B 1s core-level spectra of h-BN growth on the Ni(111) TF (see Figure S9 for XPS of the h-BN on Ni(111) SC). A large similarity can be observed for the two samples for the N 1s and B 1s core-level spectra, with a main B–N contribution in both core levels as well as an N–C contribution in the N 1s and a B–O contribution in the B 1s spectra for both samples. When HMB is deposited onto the Ni(111) surface, the N–C and B–C bonds break, leaving B and N dangling bonds that, upon temperature activation, enhance the interconnection of the HMB molecules, forming h-BN. However, as observed in the N 1s peak (Figure 2a), it has N–C contributions. This could have several explanations, for instance, the N–C bonds from the HMB did not break or, it could also be a carbon substitution of the missing B atoms to fill the B vacancies.^50–52^ Boron vacancies can occur due to various causes: i) Boron volatility in borazine-like precursors: during evaporation, HMB has to be heated fast and above >00 °C to induce the precursor to go from the solid to the gas phase. The evaporation of HMB can induce the molecules to break as observed in the mass spectra (see Figure S17), indicating the loss of B atoms.^53^ ii) The higher electronegativity of N with respect to B results in B forming weaker bonds with other elements. The high temperatures required for h-BN growth can enhance these processes and compensate for these vacancies with C forming an N–C bond that has a higher binding energy than BN.^54^ iii) Nitrogen has a higher solubility in Ni than B, which enhances N adsorption/absorption onto/into the surface with respect to B.^55^ During cooling down, the absorbed species tend to diffuse onto the surface as reported in the literature.^56,57^ The calculated atomic percentages (at %) for N and B (43% and 39%, respectively, from the XPS taken on the h-BN grown on the Ni(111) TF) are in good agreement with the processes explained above.^58^ The higher concentration of N in contrast to B could indicate the substitution with C atoms compensating for the B vacancies to maintain the honeycomb structure. The binding energies of the N 1s and B 1s core levels (397.5 and 190.5 eV, respectively, for the B–N contributions) are in good agreement with the results reported in the literature for the h-BN synthesis on Ni(111) using ammonia borane and borazine.^25,59^ C 1s, O 1s, and survey spectra are shown in Figure S10. In the work by Bachmann et al. on the synthesis of h-BN on Ni(111) surfaces, it is shown that h-BN starts forming at ≈650 K with ammonia borane and borazine being in agreement with our experimental findings.^25^

**Figure 2:**
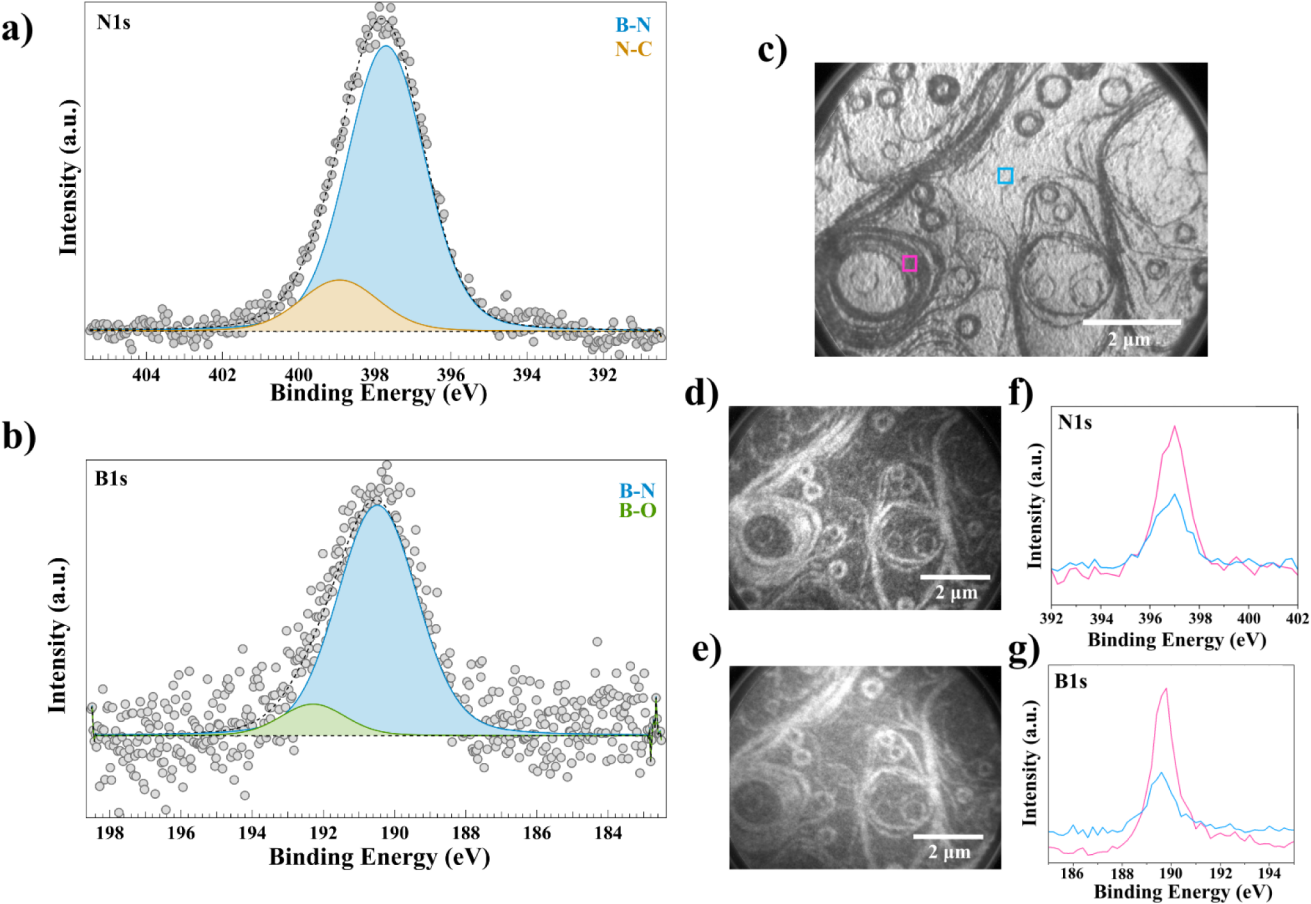
Spectroscopic characterization of h-BN on the Ni(111) TF. (a) N 1s core-level XPS spectrum. (b) B 1s core-level XPS spectrum. (c) LEEM image of the area where PEEM is performed. The pink (steps) and blue (terrace) squares indicate the two regions where the PEEM was integrated. (d) and (e) PEEM images of the N 1s (397.8 eV) and B 1s (190.4 eV) core-level regions with integrated spectra over the pink and blue regions shown in (c). (f) and (g) are the N 1s and B 1s XPS spectra extracted from the PEEM images of (d) and (e), respectively. The sky-blue line shows the XPS spectra on the terraces and the fuchsia line shows the XPS spectra on the steps.

Photoemission electron microscopy (PEEM) was performed as shown in Figure 2c on the h-BN on Ni (111) SC. This confirms the presence of h-BN on the Ni surface. Figure 2d,e shows the PEEM images collected for the N 1s and B 1s core levels, respectively. From the PEEM images, we observe that for both core levels, there is a clear difference in intensity between the steps and the terraces. This is confirmed by extracting the XPS spectra from both N 1s (Figure 2f) and B 1s (Figure 2g) core levels from the respective PEEM images, as indicated by the squares in Figure 2c. For the XPS spectra, the pink spectra show the N 1s and B 1s on the steps while the blue spectra show the N 1s and B 1s signals on the terraces. The explanation for a higher intensity of B and N at the steps could be that the steps act as nucleation sites for the molecules to anchor, dissociate, and grow the h-BN. C 1s and Ni 2p spectra are shown in Figure S11. Similar results are also observed when analyzing different PEEM data of the same area (see Figure S12).

Angle-resolved photoelectron spectroscopy (ARPES) data taken at hν = 115 eV for the clean Ni(111) SC and the h-BN are shown in Figure 3 from *E* – *E*_F_ = 1.0 to −17.0 eV. Figure 3a,b shows the energy-filtered photoelectron diffraction pattern and the projected band map from K-Γ-K (see Figure S13 cut) for the clean Ni(111) SC, while Figure 3c,d shows the data for the h-BN on the Ni(111) SC (see Figure S14 for M-Γ-M cut). Both energy-filtered diffraction patterns in Figure 3a,c are a cut of the bands in the energy at 108 eV, where the BN bands are expected. Differences are observed with a circular inner band visible in h-BN/Ni(111) that is not present for the clean Ni(111). The differences observed in Figure 3a,c are also visible when integrating the projected band map for the clean Ni(111) (Figure 3b) and the h-BN on the Ni(111) SC (Figure 3d). The h-BN valence band can be observed in Figure 3d at 108 eV (equivalent to *E* – *E*_F_ = −6 eV) at the K point. These results are in good agreement with ARPES measurements performed on h-BN on Ni(111) grown with ammonia borane and borazine.^60,61^ For a better understanding of the band structure, Figure S15 shows a detailed analysis of the h-BN on Ni(111) 2D band map.

**Figure 3:**
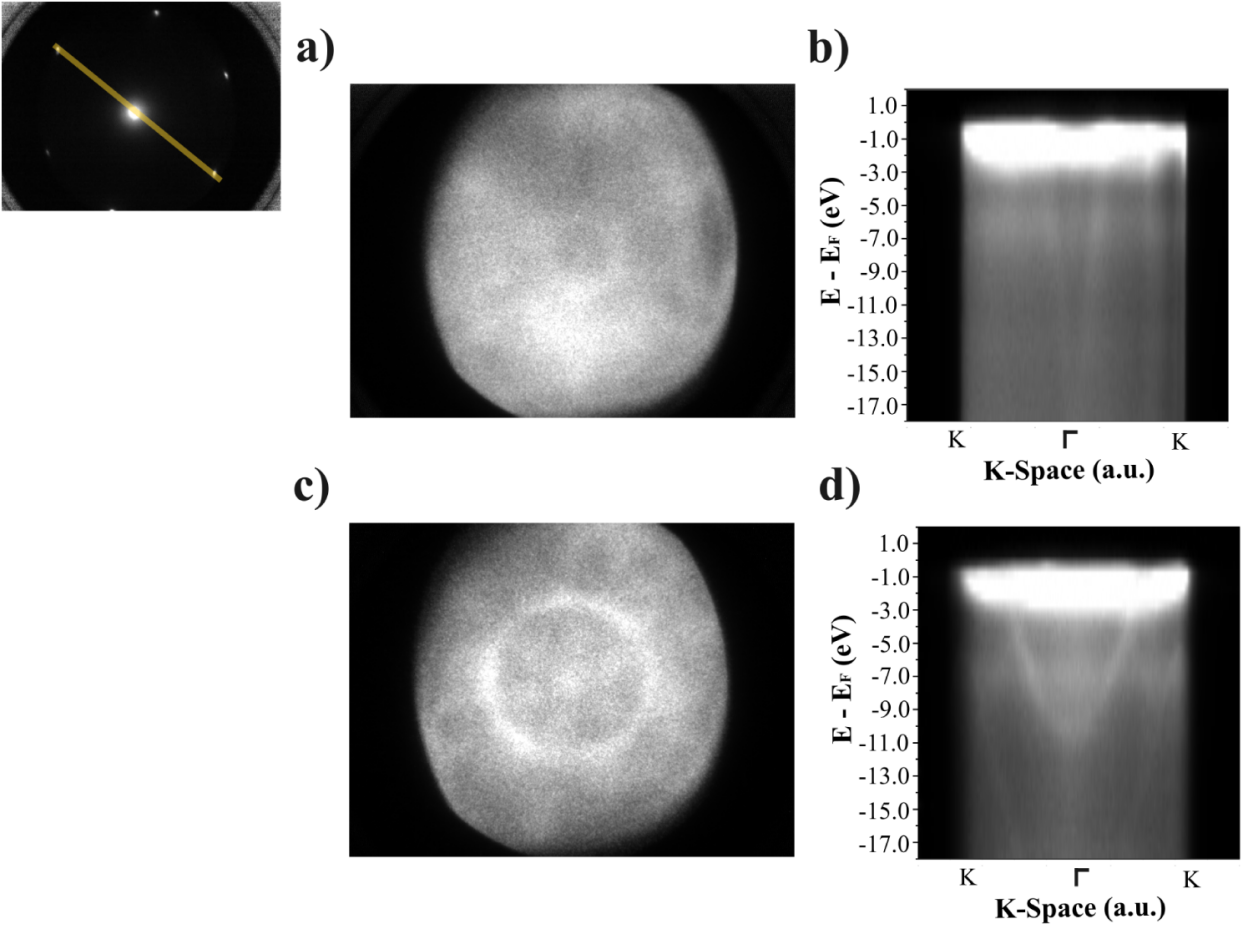
Angle-resolved photoelectron spectroscopy characterization of the clean Ni(111) and the h-BN/Ni(111) SC. Measured at hν = 115 eV. (a) Energy-filtered photoelectron diffraction pattern of Ni(111) (energy *E* = 108 eV) at 115 eV of the clean Ni(111) (see Figure S13 for ARPES on the clean Ni(111) along the M-Γ-M). (b) Projected band map along the K-Γ-K (indicated in the LEED pattern, top left). (c) Energy-filtered photoelectron diffraction pattern of h-BN/Ni(111) (energy *E* = 108 eV) at 115 eV. (d) Projected band map along the K-Γ-K direction of the h-BN Brillouin zone (see Figure S14 for ARPES on the h-BN on Ni(111) along the *M*-Γ-M).

Growing h-BN on Ni(111) SC and Ni(111) TF led to similar results. However, despite these similar findings, growing h-BN on Ni(111) TF presents some advantages compared with Ni(111) SC. Ni is known for having a high concentration of carbon in its bulk. Some studies even show the possibility of forming graphene by simply annealing a clean Ni(111) sample.^62,63^ Despite cleaning Ni, only the top layers of the sample get depleted of carbon and other impurities. While annealing to grow the h-BN, some of the carbon can diffuse onto the surface underneath the h-BN (see the C 1s PEEM in Figures S11 and S12). From the XPS spectra extracted from the PEEM image, we observe that the binding energy for the C 1s peak (≈283.4 and ≈284.0 eV) corresponds to nickel carbide (NiC).^64,65^ However, this can be remedied by using the Ni(111) TF as shown in our results, where standard cleaning procedures deplete the bulk and almost no carbon is released on the surface during the growth (Figure S10), enabling the opportunity to scale up on larger industrial wafers. Additionally, we performed Auger electron spectroscopy (AES) (Figure S16) on the h-BN grown on the Ni(111) SC at a different setup, where only B, N, and Ni peaks are visible. AES measures electrons with a larger kinetic energy, being less surface-sensitive than XPS with synchrotron energy, helping us to understand that working in a cleaner environment allows for a lower carbon concentration in the bulk of the crystal, leading to a cleaner synthesis. The pressure of the synthesis chamber at SMART (BESSY II, Germany) increased during annealing, indicating a dirtier atmosphere probably of C contained in the walls of the chamber. Additionally, the deposition was followed by mass spectrometry to confirm the evaporation of HMB. As shown in the mass spectrometry data in Figure S17, no undesired side products formed during the deposition of HMB on the Ni(111) surface. Furthermore, in contrast to the h-BN synthesis via ammonia borane or borazine, the level of formation of H_2_ is expected to be significantly lower due to the methyl groups on the edges of the HMB molecules. This can be seen in the reference for hexamethylborazine in the NIST database, in comparison for pure borazine, where the release of borazine is 14%.^66^ Additionally, the bond energies for N–H ≈ B–H < C–H, indicating that C–H bonds are stronger and less energetically favorable to be broken.^67^ Additionally, ammonia borane can decompose during evaporation into volatile compounds (NH_3_) and borazine, which are harmful for the environment.^68,69^

## Conclusions

We have demonstrated the possibility of synthesizing h-BN on two Ni(111) substrates (a single crystal and a thin film) with the same synthesis recipe. The similar results obtained for both substrates prove the perfect connection between the synthesis of 2D materials at fundamental research samples and on industrial substrates. HMB proved to be a viable precursor to grow single-crystalline h-BN at relatively low temperatures. Ni is known for having a high C solubility. From the h-BN growth on the Ni(111) SC we could observe that C had diffused from the bulk to the surface after annealing the sample. However, this issue was solved by using a Ni(111) thin film. Standard cleaning procedures are enough to deplete the bulk carbon concentration, and therefore, during annealing there is not enough C to diffuse to the surface. XPS also confirmed this; while no C signal was observed on the Ni(111) thin film, a big nickel carbide signal can be observed in the Ni(111) SC after the h-BN growth. Photoemission electron microscopy allowed for a better understanding of the growth of h-BN on the surface, showing a higher BN signal at the steps of the Ni(111) substrate.

Hexamethylborazine is a safe chemical according to its safety data sheet, and the lack of dangerous side products during evaporation as reported by the NIST database makes HMB a promising precursor for scalable h-BN synthesis on Ni(111) substrates. The reduced hydrogen production during synthesis, the safety of HMB as a precursor, the preferred growth orientation of h-BN on Ni(111), and the fact that Ni is an abundant and cheap resource make the reported synthesis an ideal procedure to be applied for industrial h-BN production.

## Supplementary Material



## Notes

Synthesis procedures of hexamethylborazine and characterization data. Additional characterization of the Ni(111) surface and h-BN on the Ni(111) TF and SC (PDF)
