# Supplementary material for: Novel Precursor for h‑BN Synthesis on Ni(111) Substrates
Source: J Phys Chem C Nanomater Interfaces. 2025 Aug 21;129(35):15693–701. doi: 10.1021/acs.jpcc.5c03822 (PMC12415827; doi:10.1021/acs.jpcc.5c03822)
Supplement: Supplementary file 1 [file jp5c03822_si_001.pdf]

Supplementary Information: Novel Precursor for h-BN Synthesis on Ni(111) Substrates

Sergi Campos-Jara,[†,‡] Tycho Roorda,[†,‡] Laurens P.M. de Jong,[‡] Vladyslav Virchenko,[‡] Andy Jiao,[‡] Mauricio J. Prieto,[¶] Liviu C. Tanase,[¶] Mohamad Mawass,[¶] Jing-Wen Hsueh,[¶] Vladimir Calvi,[‡,§] Jetse van Os,[‡] Núria Félez-Guerrero,[‡] Rick Monsma,[‡] Richard v. Rijn,[§] Thomas Schmidt,[¶] Grégory Schneider,[‡] and Irene M.N. Groot[*,‡]

†*These authors contributed equally*

‡*Leiden Insitute of Chemistry, Leiden University, Einsteinweg 55, 2333 CC Leiden, The Netherlands*

¶*Department of Interface Science, Fritz-Haber-Institut der Max-Planck-Gesellschaft, Faradayweg 4-6, Berlin, 14195, Germany*

§*Applied Nanolayers, Delft University of Technology, Feldmanweg 17, 2638 CT Delft, The Netherlands*

E-mail: i.m.n.groot@lic.leidenuniv.nl

Supplementary Information

Hexamethylborazine Synthesis

B-trichloro-N-trimethylborazine and hexamethylborazine were synthesized according to literature procedures with slight modifications (Figure S1).[1,2] Solvents were dried over 4 Å

molecular sieves. Chemical reagents were obtained from commercial suppliers and used without further purification. Nuclear Magnetic Resonance (NMR) spectra were measured on a Bruker AV-300 NMR instrument with $CDCl_3$ as internal standard (7.26 ppm in ^{1}H NMR, 77.16 ppm in ^{13}C NMR). Elemental analysis was performed by Mikroanalytisches Labor Kolbe (c/o Fraunhofer Institut UMSICHT).

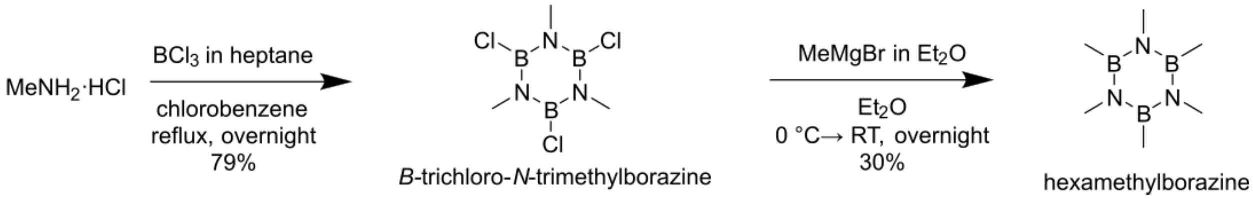

Figure S1: Synthesis scheme of hexamethylborazine.

Synthesis of B-trichloro-N-trimethylborazine

Under a N_2 atmosphere, a 3-neck Schlenk flask (500 mL) equipped with a Vigreux and a drying tube filled with $CaCl_2$ was dried with a heating gun. Then, $MeNH_2 \cdot HCl$ (11 g, 160 mmol, 1.0 eq) and dry chlorobenzene were added to the flask. BCl_3 (1.0 M in heptane, 200 mL, 200 mmol, 1.25 eq) was added dropwise over 3 hours while heating to 130 ^{o}C. After refluxing overnight, the reaction mixture was filtered over a Schlenk frit under N_2 while hot and the precipitate was rinsed with dry toluene (2 x 100 mL). The filtrate was concentrated under reduced pressure in an Ar atmosphere to obtain B-trichloro-N-trimethylborazine as pale white solids (9.5 g, 42 mmol, 79%). ^{1}H NMR (300 MHz, $CDCl_3$) δ 3.13 (s, ^{3}H). ^{13}C NMR (75 MHz, $CDCl_3$) δ 35.50.

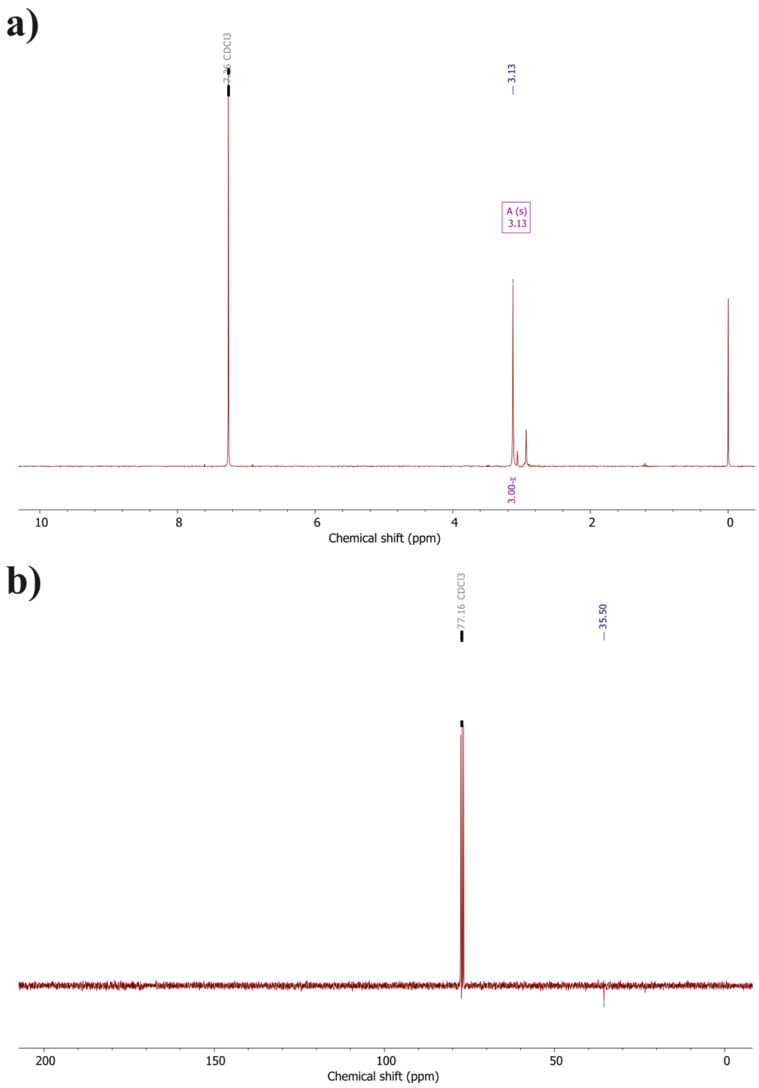

Figure S2: (a) ^{1}H NMR of B-trichloro-N-trimethylborazine in $CDCl_3$. (b) ^{13}C NMR of B-trichloro-N-trimethylborazine in $CDCl_3$.

Synthesis of Hexamethylborazine

A flame-dried 2-neck Schlenk flask (250 mL) was charged with B-trichloro-N-trimethylborazine (9.0 g, 40 mmol, 1.0 eq) and dry diethylether (50 mL) and cooled on ice. Methylmagnesium bromide (3.0 M in diethylether, 50 mL, 150 mmol, 3.75 eq) was added dropwise over 30 minutes. After stirring at room temperature overnight, the mixture was filtered and the solids were rinsed with toluene (100 mL). The organic filtrate was quenched on ice with H_2O (200 mL) while stirring, and the layers were separated in a separation funnel without additional

shaking. The organic layer was dried with Na_2SO_4, filtered and evaporated under reduced pressure to obtain pale white solids (4.5 g). The obtained solid was purified by sublimation under vacuum at 80 ^oC and collected on a cold finger at 0 ^oC. This process was repeated up to three times to obtain hexamethylborazine as white crystals (2.6 g, 16 mmol, 30%). ^1H NMR (300 MHz, $CDCl_3$) δ 2.78 (s, 3H), 0.38 (s, 3H). ^{13}C NMR (75 MHz, $CDCl_3$) δ 34.70, 25.91. ^{11}B NMR (96 MHz, $CDCl_3$) δ 36.55. Anal. Calcd for $C_6H_{18}B_3N_3$: C, 43.77; H, 11.02; N, 25.52; B, 19.7. Found: C, 43.56; H, 11.12; N, 25.34; B, 19.67.

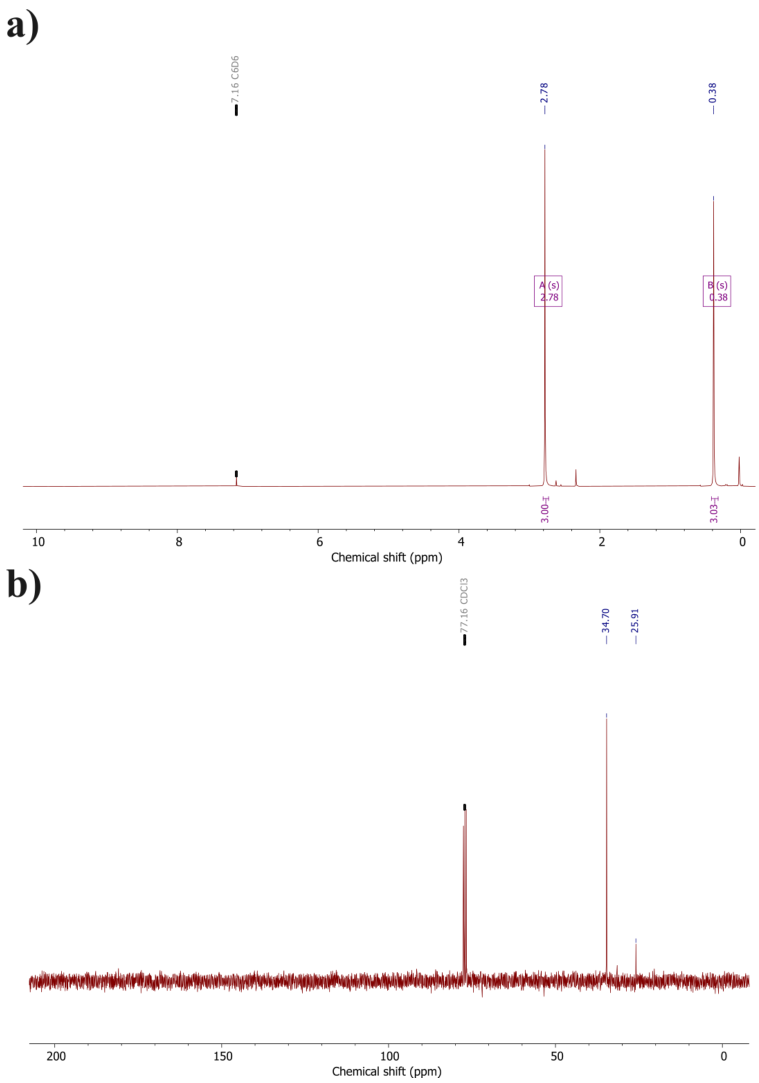

Figure S3: (a) ^1H NMR of hexamethylborazine in $CDCl_3$. (b) ^{13}C NMR of hexamethylborazine in $CDCl_3$.

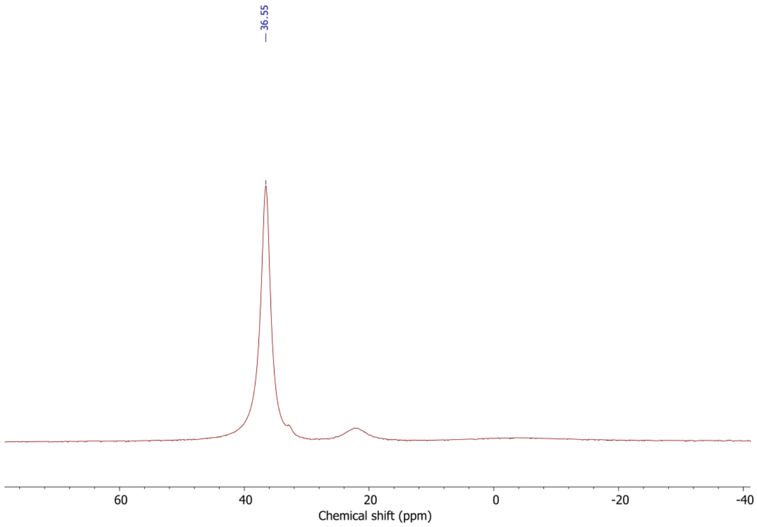

Figure S4: ^{11}B NMR of hexamethylborazine in $CDCl_3$.

Raw STM image

Scanning tunneling microscopy was performed on the h-BN grown on Ni(111) thin film (see Figure S15). Despite a blunt STM tip, we can observe features on top of the Ni(111) surface which resemble h-BN growth. The white arrow in Figure S15 points at a triangular feature growing from the Ni(111) step. Similar effects are observed in the work of Petrovic et al. where they grow h-BN on Ir(111) using borazine.[3,4]

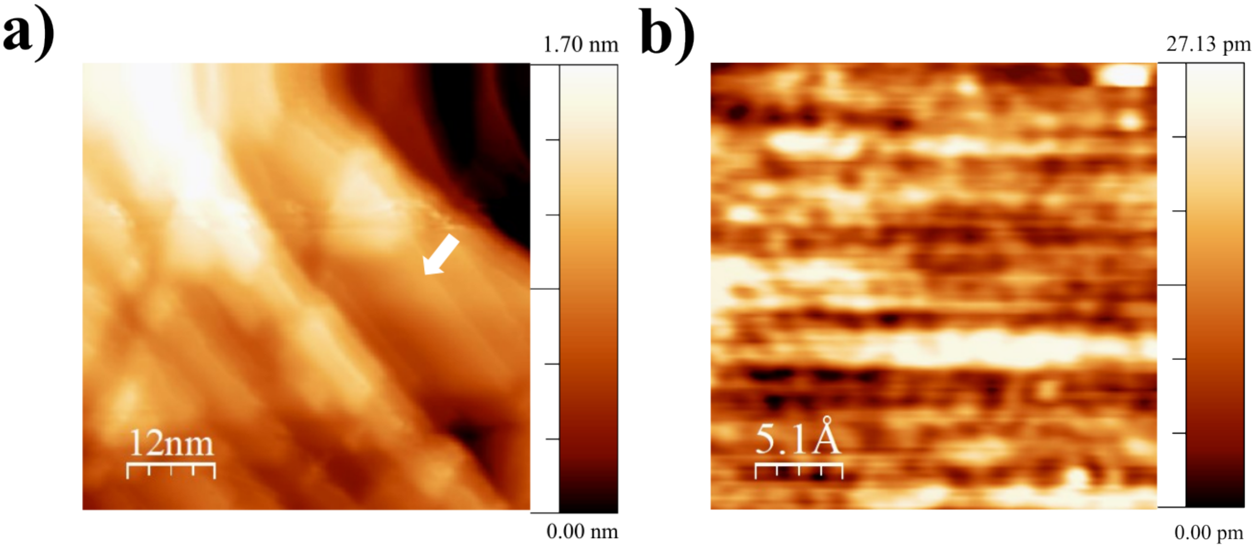

Figure S5: Scanning tunneling microscopy characterization of h-BN grown on a Ni(111) thin film. (a) Scanning tunneling microscopy image of h-BN grown on Ni(111). The white arrow points the triangular features characteristic of h-BN on the step. V_{bias} = 0.52 V, I_t = 120 pA. (b) Atomic-resolution scanning tunneling microscopy image prior to 2DFFT from the main text (Fig. 1b). V_{bias} = - 4.7 V, I_t = 65 pA.

Low Energy Electron Microscopy and Low Energy Electron Diffraction Characterization

The cleanliness of the Ni(111) single crystal surface was characterized via subsequent microscopic, diffraction, and spectroscopic techniques.

Figure S6a shows the low-energy electron microscopy (LEEM) images taken at different magnifications. The steps and terraces of Ni(111) SC are clearly visible. This is supported by the low energy electron diffraction (LEED) patterns shown in Figure S6b, where only the Ni(111) sharp spots are visible. The three-fold symmetry of the crystal could explain the difference in the brightness of the LEED spots.[5]

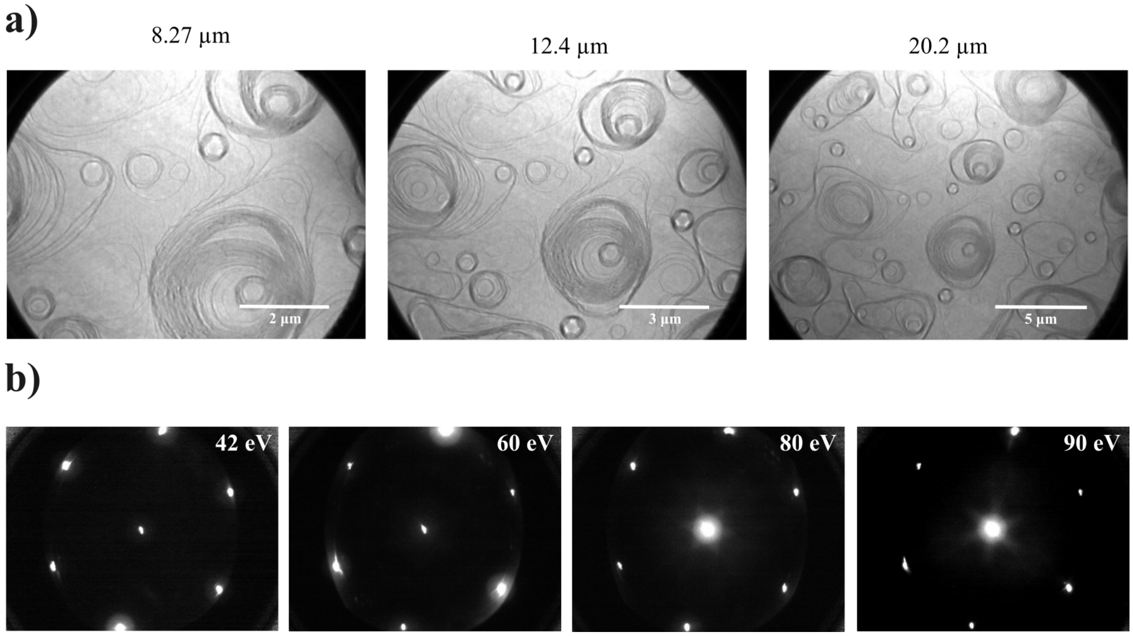

Figure S6: Characterization of the clean Ni(111) SC. (a) LEEM images of a clean surface at different lens apertures (8.27 μm, 12.4 μm, and 20.2 μm). Images were taken at 5 eV. (b) LEED patterns of the clean Ni(111) at 42, 60, 80, and 90 eV.

The h-BN grown on the Ni(111) single crystal was characterized with LEEM as shown in Figure S7a where the homogeneity of the sample is clearly visible and confirming the full coverage of the h-BN films. Despite maintaining the flatness of the surface, showing the 2D conformation of the h-BN, in contrast to the clean LEEM of the Ni(111), the surface appears rougher. The LEED results shown in S7b confirm the hypothesis that the h-BN grown on Ni(111) is single-crystalline. Since the LEED doesn't have the resolution to resolve a lattice mismatch of 0.4%, the (111) spots from the Ni(111) SC and the (111) of the single-crystalline h-BN appear overlapped.

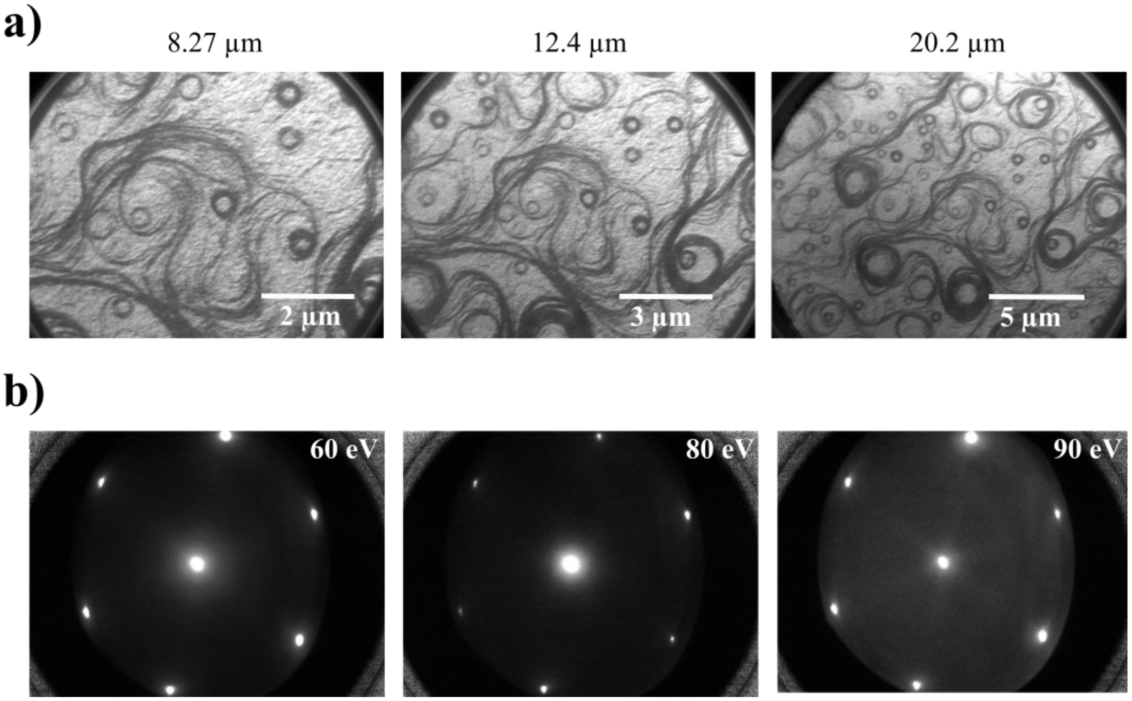

Figure S7: LEEM and LEED characterization of the h-BN grown on a Ni(111) single crystal. (a) LEEM images taken of the same area at different scales. (b) LEED patterns of the h-BN/Ni(111) single crystal taken at different energies.

Additionally, LEED was performed on the h-BN grown on the Ni(111) TF substrate, showing identical results to what is observed in the h-BN grown on Ni(111) SC.

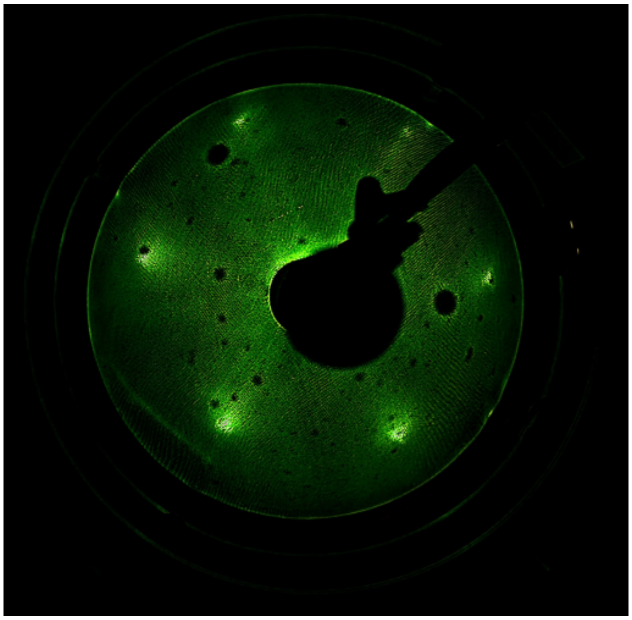

Figure S8: LEED image of the grown h-BN on Ni(111) TF at E = 90 eV.

X-Ray Photoelectron Spectroscopy Characterization

XPS characterization was performed on the different samples to confirm the presence of the different chemical species on the surface.

XPS was performed at BESSY II, Germany, on the h-BN grown on the Ni(111) single crystal. The N 1s and B 1s core level spectra shown in Fig. S9a and c are consistent with the results observed in Figure 2a and b from the main text for the h-BN on Ni(111) TF. In contrast to what is observed for the C 1s core level in Figure S9c, the concentration of carbon is higher than in Fig. S10b. However, de-convolution of the peaks confirms the larger concentration to be nickel carbide. As observed from the experiments on Ni(111) TF, the oxidation of the nickel occurs due to the water concentration in the precursor (since it has to be stored at - 20 ^{o}C until the moment of evaporation).

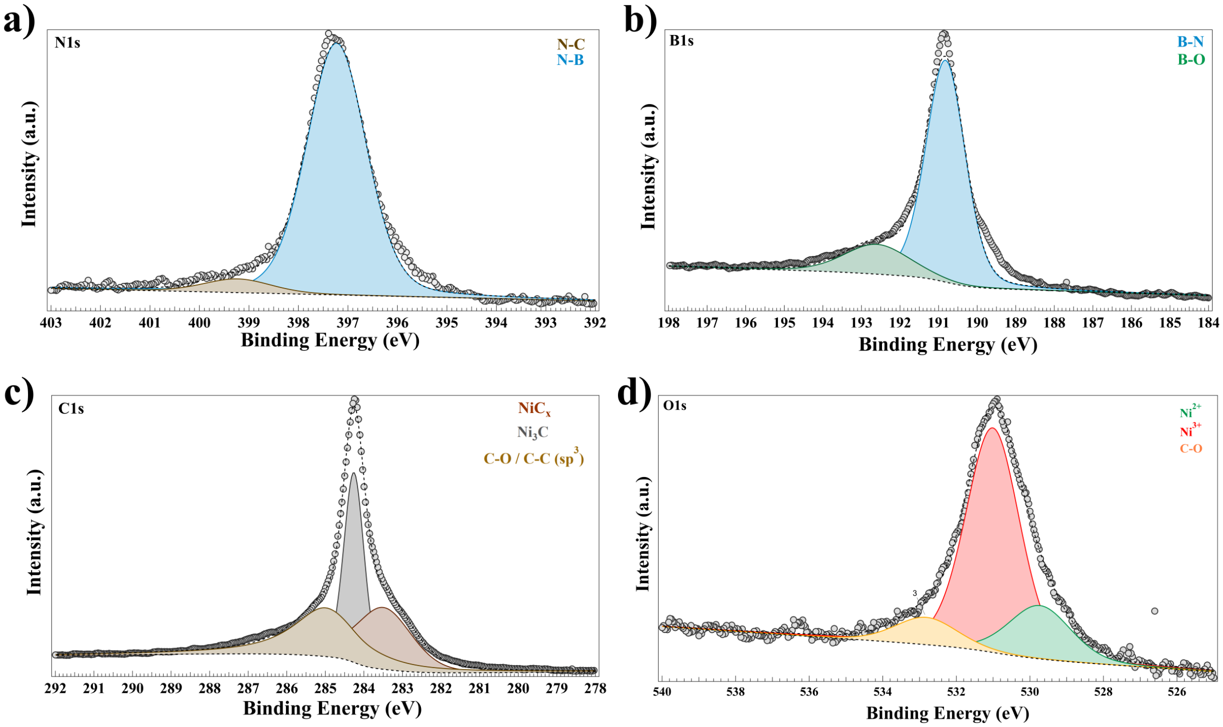

Figure S9: X-ray photoelectron spectroscopy characterization performed on h-BN on Ni(111) SC. (a) N 1s core level spectra taken at hν = 555 eV. (b) B 1s core level spectra taken at hν = 342 eV. (c) C 1s core level spectra taken at hν = 439 eV. (d) O 1s core level spectra taken at hν = 687 eV.

XPS measurements for the h-BN on Ni(111) thin film are reported in the main text. Figure S10a shows the survey spectrum after h-BN growth. Only nickel, oxygen, nitrogen, and boron are visible. Figure S10b shows the (almost negligible) concentration of carbon on the Ni(111) surface after h-BN growth. This supports the purpose of using Ni(111) thin films for h-BN growth for larger-scale synthesis. The traditional cleaning procedures are able to remove C before the h-BN growth. Since it is a few microns-thick film, the bulk is depleted of carbon and therefore, the carbon present during the synthesis diffuses into the bulk and barely diffuses onto the surface. Even though the low C1s intensity in Figure S10b makes fitting the spectrum complicated, we can get an intuition that most of the contribution of the C1s peak comes from nickel carbide and C-C bonds; additionally, a tiny C-O contribution may be observed. The O 1s contribution shown in Figure S10c can be explained by the method of evaporation. Because the precursor has to be stored at -20 ^{o}C until the

moment of evaporation, it doesn't allow for prior degassing of the crucible and precursor. As a result, when evaporating, there are still impurities in the crucible and the precursor (mainly H_2O). These impurities will be deposited, simultaneously with the precursor, on the Ni(111). Because carbon breaks off the molecule and boron is highly reactive with oxygen, this perfectly explains the species we observe in the O 1s peak. As mentioned in the main text the atomic concentrations are: B = 39.2 %, N = 43.6 %, C = 7.4 %, and O = 9.6 %. Therefore, despite the water impurities, the amount of oxygen that affects the sample is low. This could be explained by the low reactivity of h-BN with oxygen, it is formed at the time the molecules reach the surface, preventing the reaction of h-BN with the oxygen.

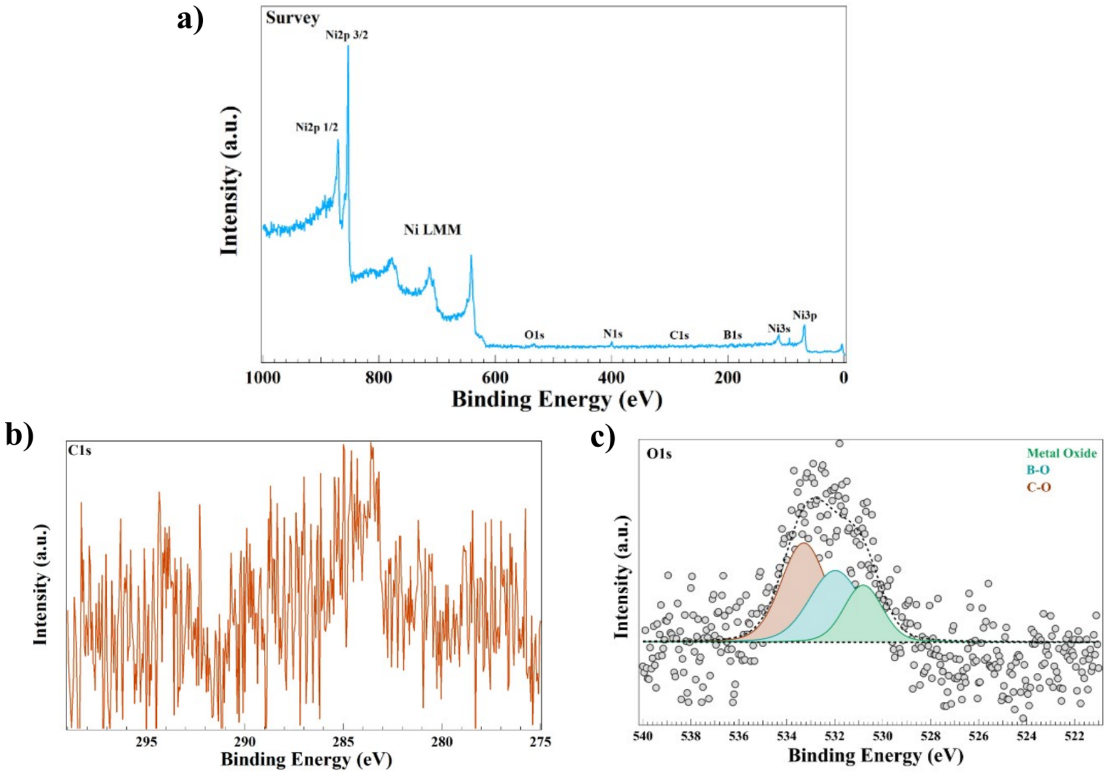

Figure S10: X-ray photoelectron spectroscopy characterization of h-BN/Ni(111) thin films. (a) Survey spectrum taken at Al Kα energy. (b) High-resolution C 1s core level spectrum. (c) High-resolution O 1s core level spectrum.

In the C 1s fitting of Figure S9c of the Ni(111) single crystal, two nickel carbide peaks are visible, and a C-C peak can also be observed. It is assumed that this peak is the convolution

of C-C (284.5 eV) and N-C (286.0 eV), and some C-O concentration (286.5 eV).

Photoemission Electron Microscopy

The C 1s and Ni 3p core level PEEM spectra of the same area as shown in the main text, Figure 2a and Figure 2b, are reported in Figure S11. In contrast to the N 1s and B 1s PEEM images shown in the main text, the C 1s core level shows a higher concentration on the terraces than on the steps. This is confirmed by the difference in the integrated areas within the same PEEM regions. The C 1s intensity on the steps (pink region) is much smaller compared to the terraces (blue region). This is in agreement with the expected results since the h-BN starts growing on the terraces, which act as the anchoring point for the molecule. However, during the cooling-down of the crystal, C impurities tend to diffuse from the bulk to the surface on Ni(111) showing a higher C intensity on the terraces. Carbon also tends to form nickel carbide which is further confirmed by Figure S9, which shows the XPS characterization performed on the sample.

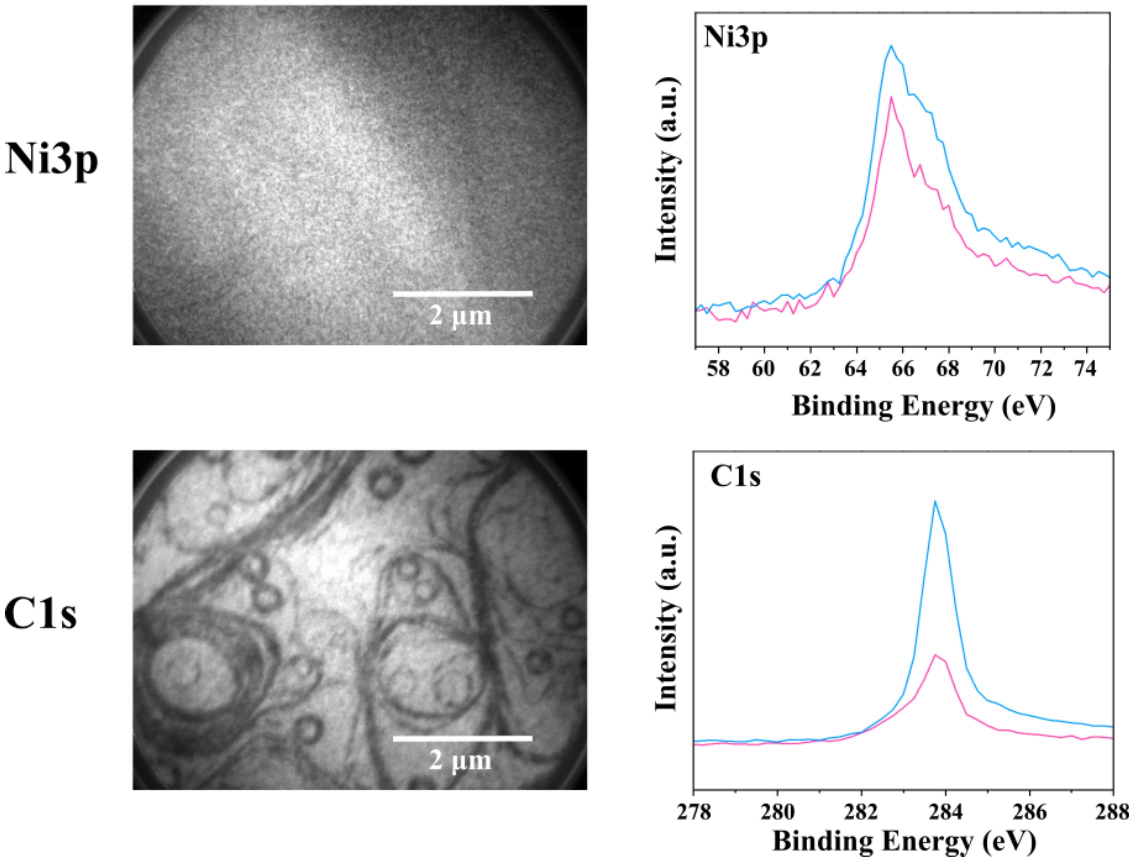

Figure S11: Photoemission electron microscopy of the Ni 3p and C 1s core level spectra over the pink and blue areas shown in the LEEM image from Figure 2 in the main text. The photon energies used were hν = 220 eV and hν = 439 eV for Ni and C, respectively.

Figure S12 shows the same PEEM performed in the same area, but integrating the steps (fuchsia) and terraces (sky blue) in a different section of the image. We observe that the results in the core levels of the different regions are very similar to the results shown above and in Figure 2. From the PEEM data we can observe that the h-BN is covering the entire Ni(111) surface. The higher N1s and B1s signal at the steps of the Ni(111) surface is expected since h-BN nucleates from the steps (like graphene). In contrast, the main C1s signal coming from the terraces leads to the assumption that this carbon originates from the Ni(111) bulk and diffuses onto the surface during the growth/cooling down. However, in situ PEEM/XPS during growth would be needed to fully confirm this hypothesis.

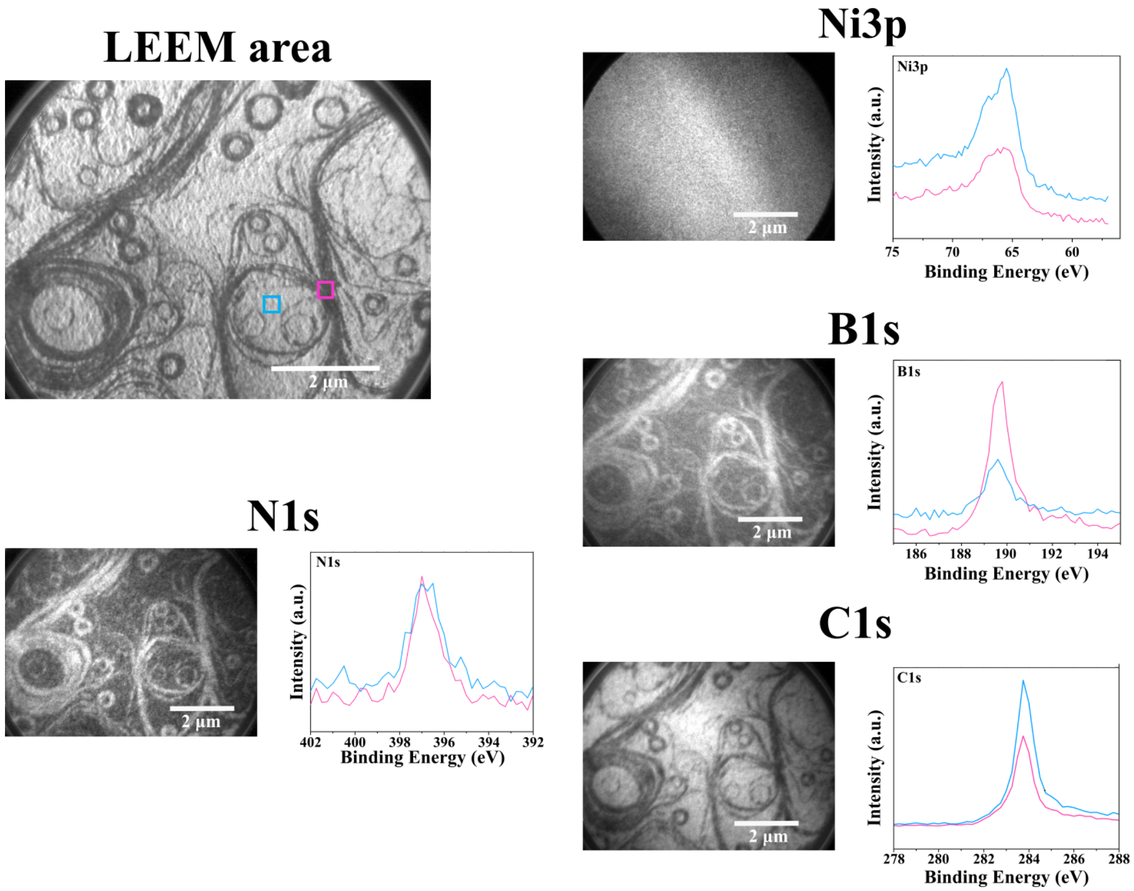

Figure S12: Photoemission electron microscopy of the Ni 3p, N 1s, B 1s, and C 1s core level spectra over the pink and sky blue boxes shown in the LEEM image. The photon energies used were hν = 555 eV, hν = 220 eV, hν = 342 eV, and hν = 439 eV for N 1s, Ni 3p, B 1s, and C 1s, respectively.

Angle-Resolved Photoelectron Specroscopy

Angle-resolved photoelectron spectroscopy (ARPES) was performed on the clean Ni(111) (see Figure S13) single crystal and h-BN grown on Ni(111) single crystal (see Figure S14).

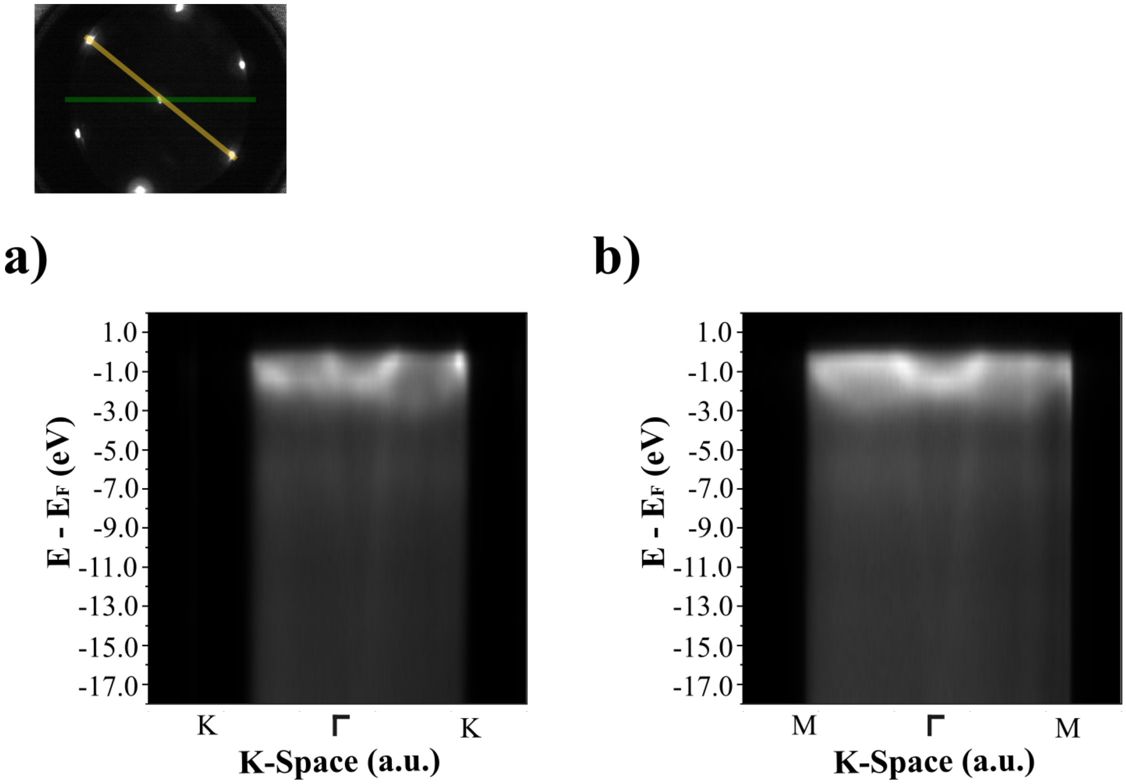

Figure S13: Angle-resolved photoelectron spectroscopy characterization of the clean Ni(111) SC surface. (a) Projected band map along the yellow cut. (b) Projected band map along the green cut. All ARPES data were taken at hν = 115 eV.

The projected band map extracted from the angle-resolved photoelectron spectroscopy measurements shown in Figure S14 match the literature results for h-BN.[6] The LEED image shows along which directions the band map was projected.

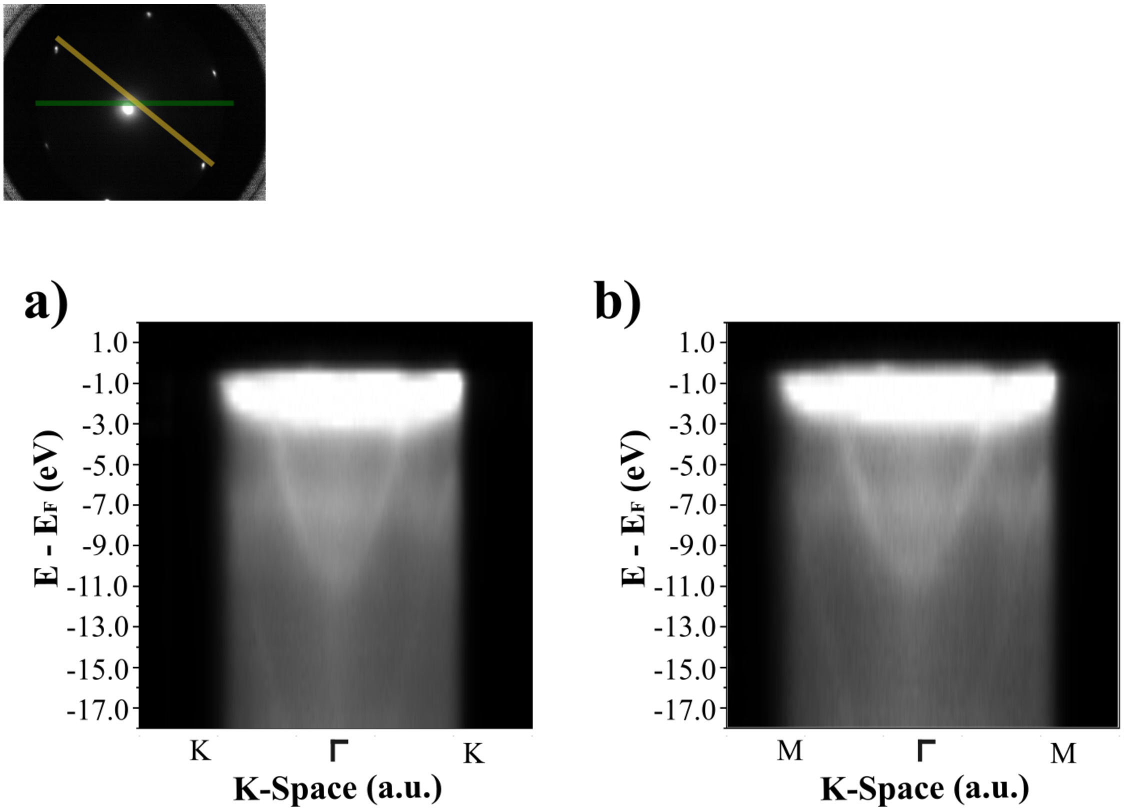

Figure S14: Angle-resolved photoelectron spectroscopy characterization of the h-BN on Ni(111) single crystal. (a) Projected band map along the yellow cut. (b) Projected band map along the green cut. All ARPES data were taken at hν = 115 eV.

To better understand the band structure of our h-BN, we have adjusted the contrast in the K-Γ-K direction to show the different bands. The valence band (indicated with a dashed line) can be observed at the K point right below the Fermi level. In contrast to most typical ARPES reported by literature,[7] we observe that the π-band from the h-BN is shifted upwards in energy, which could have several explanations. It is known that h-BN strongly interacts with Ni, which induces a hybridization of the h-BN π-bands with the Ni 3d bands.[7] In our case (see Figure S15) there is a lack of hybridization between these bands. This could most likely be explained by the formation of nickel carbide, which is less reactive than metallic nickel, inducing a similar effect as nickel oxide: quasi-free-standing h-BN on Ni.[8] This would induce a weaker interaction between the h-BN and Ni and therefore no hybridization is observed in Figure S15. The shift in energy of the h-BN πands could be explained by this

lack of hybridization, but also because of the C atoms substituting B vacancies giving an extra electron to the system compared to B, resulting in shifting in energy of the bands,[9] altogether explaining the main differences.

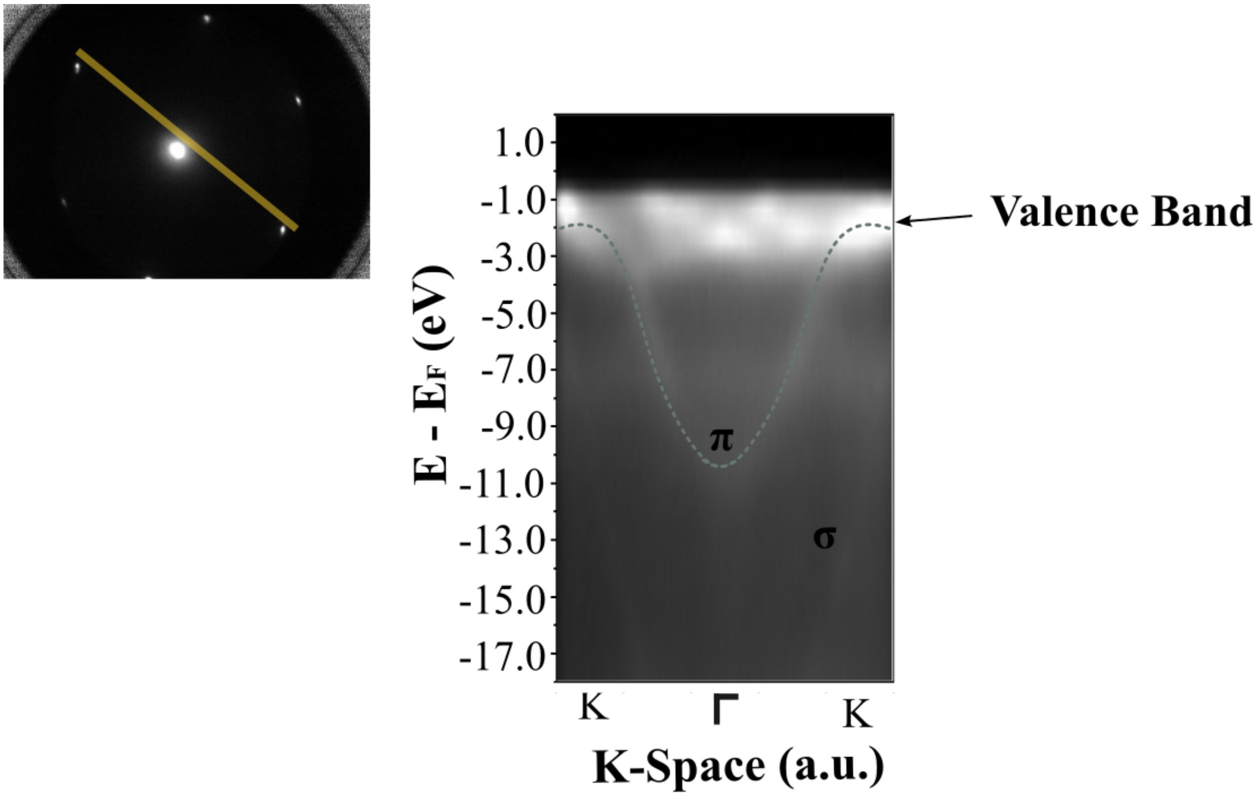

Figure S15: Angle-resolved photoelectron spectroscopy characterization of the h-BN on Ni(111) single crystal along the K-Γ-K cut indicated in the LEED with adjusted contrast for a better identification of the bands.

Additional Characterization

h-BN was synthesized on top of Ni(111) SC at our facilities and Auger electron spectroscopy (AES) was performed to understand the presence of C on the single crystal. However, as seen in S16, after performing the synthesis under the exact same conditions, the AES spectrum shows a clear B, N, and Ni peak as expected from the synthesis, but no C signal. This is in good correlation with the results reported by Harpale et al., where they study the binding energy of a C-C bond on a Ni and Cu substrate as well as the binding energy of C atoms

diffusing into these substrates during graphene growth.[10] On Cu the binding energy of C-C coupling is more favorable than the bulk diffusion of C in Cu's bulk, for Ni it is the opposite scenario is true.

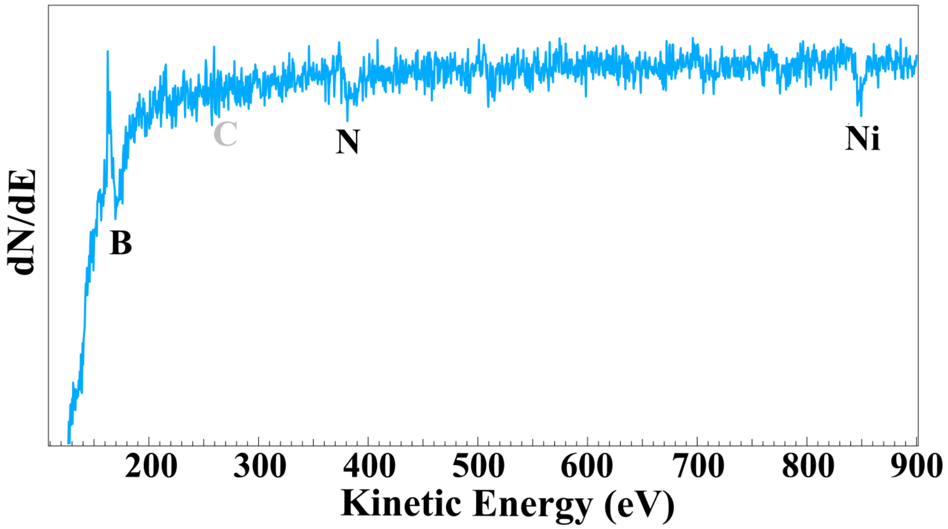

Figure S16: Auger electron spectroscopy of h-BN grown on Ni(111) SC performed at 2000 eV. Clear B, N, and Ni peaks are visible.

During evaporation, the molecule was followed with a quadrupole mass spectrometer (QMS) as shown in Figure S17a. When comparing with the NIST reference (see Figure S17b), we can confirm the presence of the expected molecule fragments in the chamber and, therefore, the successful evaporation of HMB.

The HMB precursor is contained in a stainless steel vial connected to a leak valve and pumped down to around 10^{-6} mbar. The vial is covered by a dewar flask filled with dry ice to maintain it below -20 ^{o}C until the moment of evaporation. Despite the baking out of the gas lines, the vial can't be baked nor can the precursor be degassed, as it decomposes at RT. As a result, we observe high-intensity peaks of H_2, H_2O and hydrocarbons.

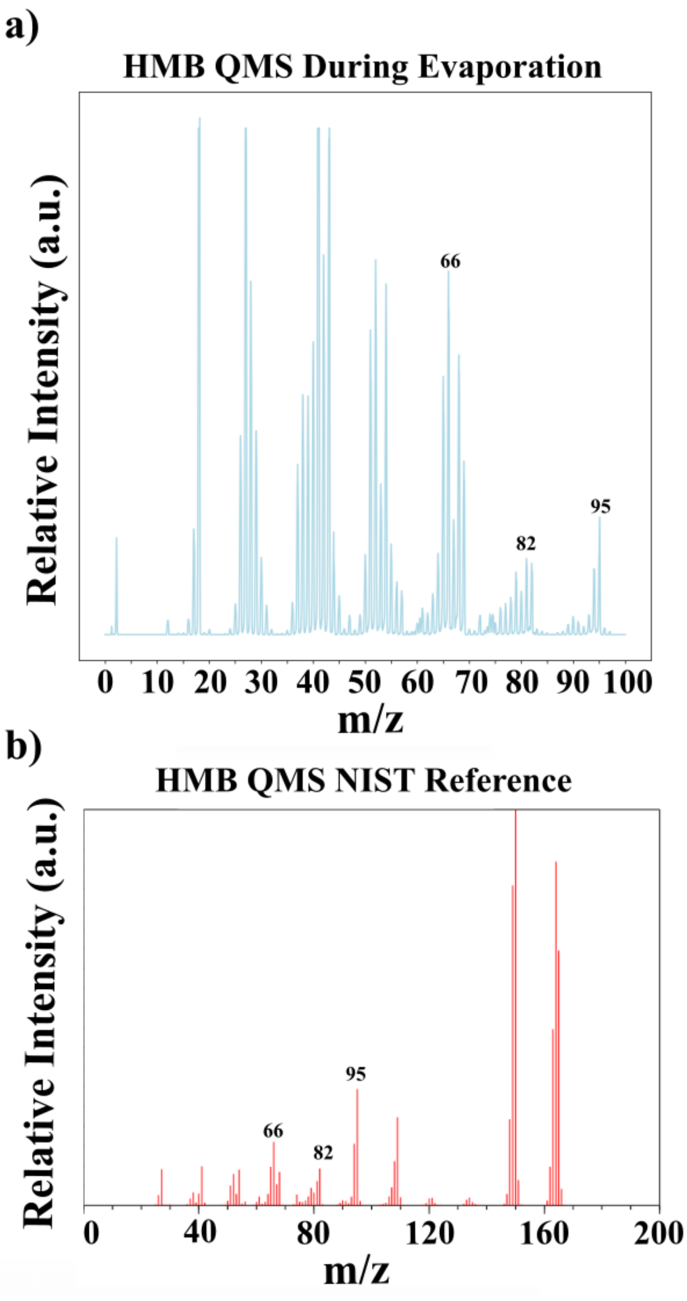

H

Figure S17: QMS reults of hexamethylborazine. (a) Mass spectrum taken during HMB deposition on Ni(111). (b) Reference mass spectrometry data extracted from NIST.[11] The highest intensity of HMB masses below m/z = 100 are indicated on both figures.

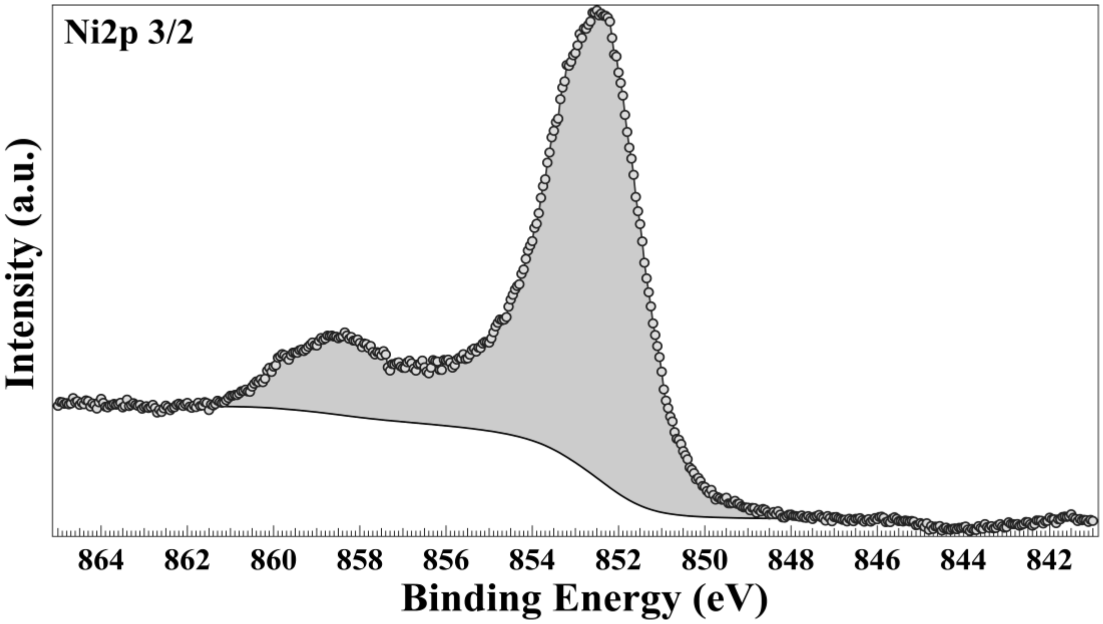

Figure S18: High-resolution Ni2p 3/2 spectrum of the clean Ni(111) thin film. hν = 1486.4 eV.

References

(1) Hohnstedt, L. F.; Haworth, D. T. Preparation of N-Trisubstituted Borazines by Reduction of B-Trichloroborazines. *Journal of the American Chemical Society* **1960**, *82*, 89–92.

(2) Haworth, D. T.; Hohnstedt, L. F. Synthesis of B-Trisubstituted Borazines by Reaction of B-Trichloroborazine with Grignard Reagents. *Journal of the American Chemical Society* **1960**, *82*, 3860–3862.

(3) Petrović, M.; Hagemann, U.; von Hoegen, M. H.; zu Heringdorf, F. J. M. Microanalysis of single-layer hexagonal boron nitride islands on Ir(111). *Applied Surface Science* **2017**, *420*, 504–510.

(4) Petrović, M.; von Hoegen, M. H.; zu Heringdorf, F. J. M. Equilibrium shape of single-layer hexagonal boron nitride islands on iridium. *Scientific Reports* **2019**, *9*, 1–6.

(5) Dahlgren, D.; Hemminger, J. C. Symmetry extinction of LEED beams for naphthalene adsorbed on Pt(111). *Surface Science* **1981**, *109*, L513–L518.

(6) Verbitskiy, N. I.; Fedorov, A. V.; Profeta, G.; Stroppa, A.; Petaccia, L.; Senkovskiy, B.; Nefedov, A.; Wöll, C.; Usachov, D. Y.; Vyalikh, D. V. et al. Atomically precise semiconductor—graphene and hBN interfaces by Ge intercalation. *Scientific Reports* **2015**, *5*, 1–9.

(7) Usachov, D.; Adamchuk, V. K.; Haberer, D.; Grüneis, A.; Sachdev, H.; Preobrajenski, A. B.; Laubschat, C.; Vyalikh, D. V. Quasifreestanding single-layer hexagonal boron nitride as a substrate for graphene synthesis. *Physical Review B - Condensed Matter and Materials Physics* **2010**, *82*, 075415.

(8) Suzuki, S.; Haruyama, Y.; Niibe, M.; Tokushima, T.; Yamaguchi, A.; Utsumi, Y.; Ito, A.; Kadowaki, R.; Maruta, A.; Abukawa, T. Quasi-free-standing monolayer hexagonal boron nitride on Ni. *Materials Research Express* **2018**, *6*, 016304.

(9) Gupta, S. K.; He, H.; Lukačević, I.; Pandey, R. Spin-dependent electron transport in C and Ge doped BN monolayers. *Physical Chemistry Chemical Physics* **2017**, *19*, 30370–30380.

(10) Harpale, A.; Panesi, M.; Chew, H. B. Communication: Surface-to-bulk diffusion of isolated versus interacting C atoms in Ni(111) and Cu(111) substrates: A first principle investigation. *Journal of Chemical Physics* **2015**, *142*, 42.

(11) NIST Borazine,hexamethyl-. `https://webbook.nist.gov/cgi/inchi?ID=C877076&Mask=200#Mass-Spec`, Accessed: 2024-10-11.
